# Laparoscopic Versus Open Surgery for Penetrating and Blunt Abdominal Trauma: A Systematic Review and Meta-Analysis

**DOI:** 10.7759/cureus.106951

**Published:** 2026-04-13

**Authors:** Abdulla Muhanna, Wajdy Suliman, Khalid B Mohammed, Ashraf Muhammad, Vinan Elhassan, Obay Abdulmgid, Meshael Alghaihab, Aref Dayl, Mohamed Ali, Mohammad AbdelMoaty, Ali Alkasi, Ali Alahmre, Bayan Alabed, Ali S Metwaly

**Affiliations:** 1 General and Bariatric Surgery, King Fahad Hospital - Jeddah, Jeddah, SAU; 2 General Surgery, GNP Hospital, Jeddah, SAU; 3 General Surgery, International Medical Center Hospital, Jeddah, SAU; 4 Surgery, Saudi Arabia Ministry of Health, Al Baha, SAU; 5 College of Medicine and Surgery, Shendi University, Riyadh, SAU; 6 Faculty of Medicine, Alexandria University, Alexandria, EGY; 7 General Surgery, King Saud bin Abdulaziz University for Health Sciences, Riyadh, SAU; 8 Medical School, University of Science and Technology, Sana’a, YEM; 9 Pediatric Surgery, El Sheikh Zayed Specialized Hospital, Giza, EGY; 10 General Surgery, Armed Forces Hospital Southern Region, Khamis Mushait, SAU; 11 General Surgery, College of Medicine, King Khalid University, Abha, SAU; 12 General Surgery, King Saud Medical City, Riyadh, SAU; 13 Medical Internship Program, Eastern Health Cluster, Riffa, BHR; 14 Precision Medicine, Faculty of Pharmacy, Alexandria University, Alexandria, EGY

**Keywords:** abdominal trauma, blunt trauma, evidence-based medicine, laparoscopy, laparotomy, meta-analysis, minimally invasive surgery, penetrating trauma, surgical outcomes

## Abstract

Exploratory laparotomy (EL) is the standard approach for managing abdominal trauma; however, it is associated with significant morbidity and high rates of non-therapeutic interventions. While laparoscopy is utilized to mitigate these risks, its therapeutic role and comparative safety profile, particularly in blunt trauma, remain controversial. This systematic review and meta-analysis compared the clinical outcomes of laparoscopic versus open surgery in hemodynamically stable patients with penetrating and blunt abdominal trauma.

A systematic search of MEDLINE, Embase, CENTRAL, CINAHL, and Scopus was conducted from January 1990 to February 2026, adhering to PRISMA 2020 guidelines (PROSPERO: CRD420261296724). Comparative studies (randomized controlled trials and observational cohorts) evaluating laparoscopy versus open laparotomy in adult and pediatric trauma patients were included. The primary outcomes were mortality and length of hospital stay (LOS); secondary outcomes included overall complications and conversion rates. Random-effects models with Hartung-Knapp-Sidik-Jonkman (HKSJ) adjustments were used for pooling. Subgroup analysis, meta-regression, and Grading of Recommendations Assessment, Development, and Evaluation (GRADE) assessments were performed. Fifteen studies comprising 22,242 patients (3,965 laparoscopic and 18,277 open) were included.

The laparoscopic approach was associated with a significant reduction in hospital LOS (mean difference (MD) -3.55 days; 95% confidence interval (CI): -4.92 to -2.18; p < 0.001; GRADE: moderate). Patients undergoing laparoscopy demonstrated 52% lower odds of mortality (odds ratio (OR) 0.48; 95% CI: 0.18 to 1.30; p = 0.12; GRADE: low) and a 55% reduction in overall postoperative complications (OR 0.45; 95% CI: 0.34 to 0.60; p < 0.001; GRADE: low), although these findings were susceptible to selection bias. Subgroup analyses confirmed that the reduction in LOS remained significant across both penetrating and blunt injury mechanisms. The pooled conversion rate from laparoscopy to open surgery was 18.6%. Leave-one-out sensitivity analyses and cumulative meta-analysis demonstrated the robustness and temporal stability of the treatment effect.

In appropriately selected, hemodynamically stable patients with abdominal trauma, laparoscopy is a safe and highly effective alternative to open laparotomy. It significantly reduces hospital LOS and postoperative morbidity without increasing mortality, proving efficacious for both penetrating and blunt injury mechanisms. These findings support the integration of a laparoscopic-first approach as a standard of care in modern trauma algorithms.

## Introduction and background

Abdominal trauma, encompassing both blunt and penetrating mechanisms, accounts for a significant proportion of trauma-related morbidity and mortality worldwide [1,2]. Exploratory laparotomy (EL) is the gold standard for the evaluation and management of intra-abdominal injuries [3,4]. While EL remains a lifesaving and mandatory intervention for hemodynamically unstable patients or those with catastrophic multi-organ injuries, the mandate for routine laparotomy results in a high incidence of negative or non-therapeutic laparotomies (NTLs) [1,3]. NTL rates can reach as high as 40%-61% in mandatory exploration protocols [2,5]. A negative or NTL is far from a benign intervention, as it exposes patients to the physiological stress of a major laparotomy and carries a complication rate of up to 20%-33%. These complications include surgical site infections (SSIs), pneumonia, prolonged postoperative ileus, and the long-term risks of adhesive small bowel obstruction and ventral hernia formation [1,4,6].

Over the past three decades, acute care surgery has shifted toward minimally invasive techniques to mitigate iatrogenic morbidity associated with open surgery [7,8]. The application of laparoscopy has expanded as it is introduced as a purely diagnostic tool to rule out peritoneal violation or occult diaphragmatic tears in penetrating trauma [2,9]. Driven by advancements in high-definition imaging, laparoscopic instrumentation, and growing surgical expertise, trauma laparoscopy is utilized for therapeutic purposes, as it has proven feasible for definitive interventions such as diaphragmatic repair, solid organ hemostasis, and hollow viscus repair in both blunt and penetrating abdominal trauma [8,10]. Reflecting this shift, the World Society of Emergency Surgery (WSES) endorsed the laparoscopic-first approach for hemodynamically stable patients requiring emergency abdominal surgery [7].

The literature suggests that, compared to conventional open surgery, a diagnostic laparoscopy-and-proceed approach offers clinical benefits for appropriately selected hemodynamically stable trauma patients [4,11]. Previous meta-analyses have demonstrated that laparoscopy is associated with a significant reduction in postoperative morbidity, specifically SSIs and pulmonary complications, as well as shorter hospital and intensive care unit (ICU) LOS and an accelerated return of bowel function [1,4,5]. Furthermore, laparoscopy functions as an effective triage tool, safely avoiding the morbidity of an open procedure in patients without injuries requiring surgical repair [1,5].

Despite these compelling advantages, the adoption of laparoscopy in trauma algorithms remains highly heterogeneous [1,7]. Skepticism persists within the trauma community regarding the risk of missed injuries due to the absence of tactile feedback, particularly subtle hollow viscus perforations, and the potential for life-threatening physiological derangements associated with pneumoperitoneum in acutely injured patients [2,8]. Furthermore, the evidence base is highly fragmented; prior systematic reviews are often limited by the inclusion of small, single-center retrospective cohorts, a high degree of methodological heterogeneity, or a narrow focus isolating either blunt or penetrating mechanisms without providing a comprehensive overview of modern trauma practice [4,10,12].

Given the evolution of minimally invasive surgical (MIS) techniques and the recent influx of clinical data, a contemporary and rigorous synthesis of the evidence is imperative. Therefore, the primary objective of this systematic review and meta-analysis was to evaluate the comparative effectiveness and safety of laparoscopic versus open surgical approaches in patients with penetrating and blunt abdominal trauma. By clarifying differences in perioperative mortality, overall morbidity, operative outcomes, and recovery metrics, this study aimed to provide evidence-based guidance to optimize surgical decision-making and improve clinical outcomes in modern trauma care.

## Review

Methods

Protocol and Registration

The design and execution of this quantitative synthesis followed the guidelines set forth by the 2020 Preferred Reporting Items for Systematic Reviews and Meta-Analyses (PRISMA) statement [13]. Furthermore, the methodological framework was prospectively registered with the International Prospective Register of Systematic Reviews database (PROSPERO identifier: CRD420261296724).

Search Strategy and Selection Criteria

A search of electronic literature was carried out across multiple databases, including Scopus, CINAHL, the Cochrane Central Register of Controlled Trials (CENTRAL), Embase (via Ovid), and MEDLINE (via PubMed), spanning from January 1990 to February 2026. The search syntax relied on a strategic combination of free-text keywords and Medical Subject Headings (MeSH) pertinent to “laparotomy,” “minimally invasive surgery,” “laparoscopy,” “penetrating trauma,” “blunt trauma,” and “abdominal trauma.” To ensure comprehensive coverage, the reference bibliographies of all included primary studies and relevant review articles were manually scrutinized to locate additional qualifying reports.

Studies were included if they met the following criteria: (1) randomized controlled trials (RCTs) or non-randomized comparative studies (prospective or retrospective cohorts, case-control studies); (2) adult and pediatric populations presenting with penetrating or blunt abdominal trauma; and (3) a direct comparison of clinical outcomes between laparoscopic and open surgical approaches. Non-comparative case series, case reports, animal studies, and conference abstracts lacking full methodological data were excluded. Two independent investigators screened the titles, abstracts, and full texts. Inter-rater reliability during the screening and selection processes was quantified using Cohen’s kappa statistic [14], and disagreements were resolved via adjudication by a third investigator.

Risk of Bias and Methodological Quality Assessment

Methodological quality and risk of bias were independently appraised by two investigators using tools tailored to the study design. RCTs were evaluated using the Cochrane Risk of Bias 2 (RoB 2) tool [15], assessing biases arising from the randomization process, deviations from intended interventions, missing outcome data, measurement of the outcome, and selection of the reported result. Non-randomized observational studies were critically appraised utilizing the Risk of Bias in Non-randomized Studies of Interventions (ROBINS-I) tool [16]. Additionally, matched and unmatched retrospective cohort studies were evaluated using the Newcastle-Ottawa Scale (NOS) [17], classifying study quality based on selection, comparability, and outcome ascertainment.

Statistical Modeling and Effect Measures

All statistical analyses and meta-analytic modeling were executed in the R programming environment (version 4.5.2; R Foundation for Statistical Computing, Vienna, Austria) [18], utilizing the meta and metafor packages [19,20]. For dichotomous outcomes (e.g., mortality, postoperative complications, conversion rates), effect sizes were pooled as odds ratios (ORs) and risk ratios (RRs). For continuous outcomes (e.g., operative time, length of hospital stay (LOS)), mean differences (MDs) and standardized mean differences (SMDs) were calculated.

Due to the anticipated clinical and methodological heterogeneity in trauma populations and varied institutional settings, pooling was conducted using a random-effects model. The DerSimonian-Laird estimator was applied as the primary pooling method [21], whereas restricted maximum likelihood (REML) estimation was utilized for continuous outcome estimators to optimize variance assessment [22]. To mitigate the risk of Type I errors and produce more conservative, reliable variance estimates, particularly when pooling a moderate number of studies, the Hartung-Knapp-Sidik-Jonkman (HKSJ) adjustment was implemented [23]. Results are presented with 95% confidence intervals (CIs). Furthermore, 95% prediction intervals (PIs) were calculated to estimate the dispersion of true effects and provide the expected range of treatment effects in future clinical settings [24].

Assessment of Heterogeneity and Robustness

Statistical heterogeneity (dispersion and inconsistency) among the included studies was evaluated using Cochran’s Q test (with statistical significance defined as p < 0.10) and quantified using the I² statistic, where values of 25%, 50%, and 75% indicated low, moderate, and high heterogeneity, respectively [25].

To explore potential sources of heterogeneity, moderator analyses were conducted using meta-regression and subgroup analyses. The pre-defined subgroups included the mechanism of injury (blunt vs. penetrating), study design (RCT vs. observational), and patient hemodynamic status. The robustness of the pooled estimates was further tested via comprehensive adjustment and sensitivity analyses, including leave-one-out influence analyses and the exclusion of studies deemed to have a critical or serious risk of bias. Additionally, a cumulative meta-analysis was performed to track the temporal evolution of the pooled effect sizes and ascertain how the maturation of laparoscopic techniques over time has influenced clinical outcomes [26].

Publication Bias and Small-Study Effects

Reporting and dissemination biases were assessed when 10 or more studies were available for a given outcome. Visual assessment of bias was conducted using contour-enhanced funnel plots [27]. To formally evaluate small-study effects and funnel plot asymmetry, statistical tests were employed: Egger’s regression test [28] was used for continuous outcomes, whereas Harbord’s modified test [29] or Peters’ test [30] was applied for dichotomous outcomes to avoid mathematical artefacts common with sparse event rates.

Certainty of Evidence

The overall certainty and strength of the evidence for each primary and secondary outcome were evaluated using the Grading of Recommendations Assessment, Development, and Evaluation (GRADE) framework [31]. Evidence was graded as high, moderate, low, or very low based on five domains: overall risk of bias, inconsistency of results, indirectness of evidence, imprecision of effect estimates, and publication bias.

Results

Study Selection and Characteristics

**Figure 1:**
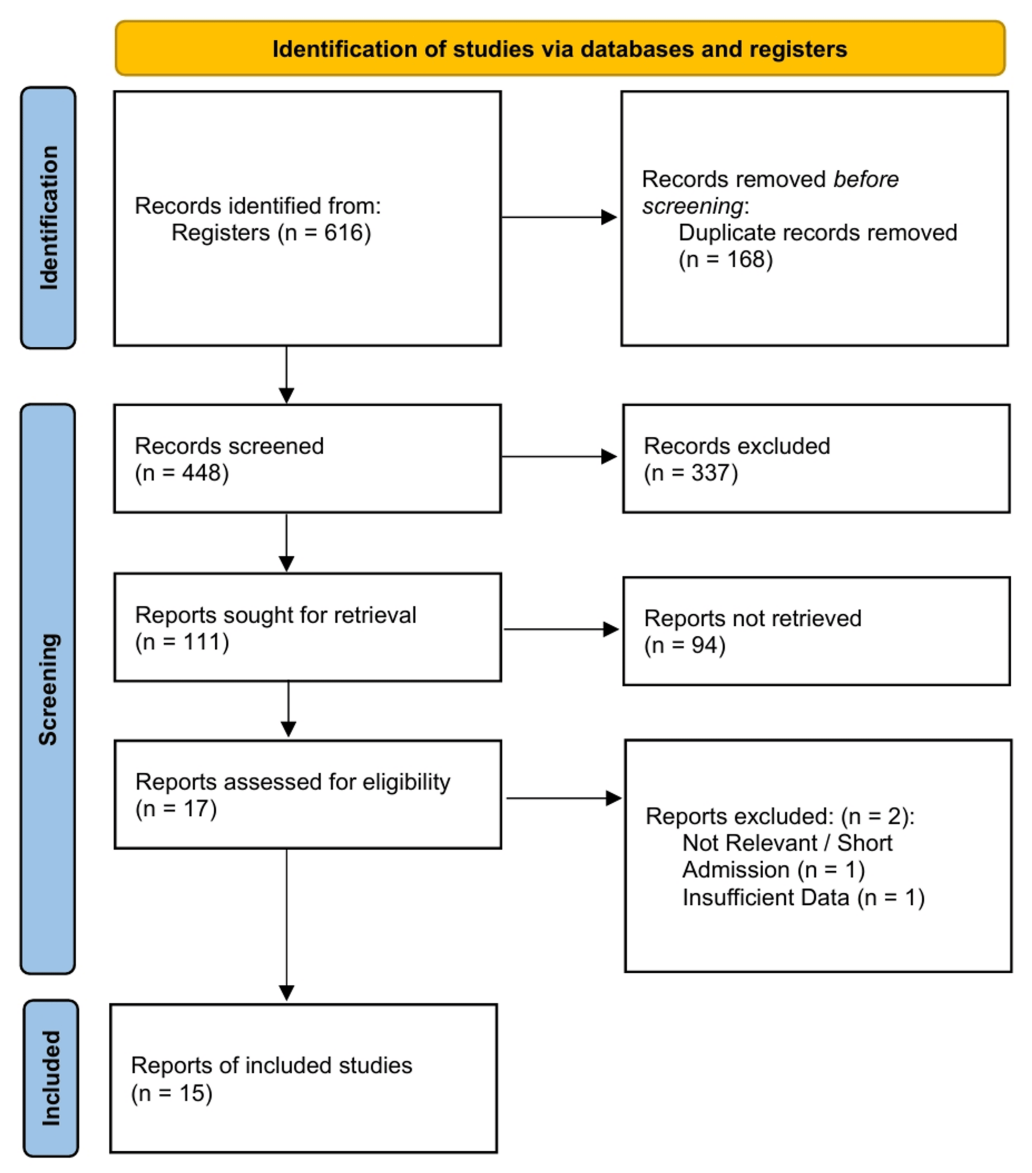
PRISMA 2020 flow diagram of study selection process.

The literature search yielded 616 records. After removing duplicates and screening titles and abstracts, 17 full-text articles were assessed for eligibility. Two studies were excluded due to insufficient data or short admission times, leaving 15 studies for inclusion in the final quantitative synthesis [32-46]. The PRISMA flow diagram details the selection process (Figure 1).

The final cohort comprised 22,242 patients; 3,965 (17.8%) underwent laparoscopic surgery, and 18,277 (82.2%) underwent open laparotomy. The included studies consisted of one RCT [36] and 14 observational studies (prospective/retrospective cohorts) published between 2000 and 2025. The mechanism of trauma varied across studies: five focused on penetrating trauma [36,37,39,43,45], four on blunt trauma [32,41,42,46], and six included a mixed population [33-35,38,40,44]. Patient demographics, injury severity, and hemodynamic stability protocols were comparable across the matched cohorts (Table 1).

**Table 1:** Characteristics of included studies (N = 15). *Indicates that the original study reported non-parametric data (median and interquartile range or range). The mean ± SD has been mathematically estimated for standard harmonization using the methods of Wan et al. (2022) [4] and Luo et al. (2018) [47]. TQIP, Trauma Quality Improvement Program; NTDB, National Trauma Data Bank; GU, Genitourinary; LOS, Length of Stay; EBL, Estimated Blood Loss; Op Time, Operative Time; NOS, Newcastle-Ottawa Scale; RoB 2, Cochrane Risk of Bias Tool 2; ROBINS-I, Risk Of Bias In Non-randomized Studies - of Interventions; NR, Not Reported

Study	Year	Country	Study Design	Trauma Type	Population (N)	Intervention (Lap vs Open)	Mean Age ± SD (Year) (Lap vs Open)	Key Outcomes Reported	Quality (Tool)
Erwin et al. [32]	2025	USA	Retrospective Cohort (TQIP)	Blunt	13,748	1,659 vs 12,089	36.0 ± 21.4* vs 36.0 ± 20.0*	Mortality, LOS, Complications	Moderate (ROBINS-I)
Swendiman et al. [33]	2019	USA	Retrospective Cohort (NTDB)	Mixed (Pediatric)	4,554	355 vs 4,199	9.0 ± 3.9 vs 8.3 ± 4.2	Mortality, LOS, Conversion	Moderate (ROBINS-I)
Trejo-Ávila et al. [34]	2017	Mexico	Case-Matched Cohort	Mixed	38	19 vs 19	25.5 ± 7.7 vs 30.9 ± 10.9	Operative Time, LOS, EBL	High (NOS: 8/9)
Collins et al. [35]	2022	USA	Case-Matched Cohort (TQIP)	Mixed (GU)	384	192 vs 192	34.0 ± 17.0 vs 37.0 ± 13.0	Mortality, Complications, LOS	High (NOS: 8/9)
Leppäniemi et al. [36]	2003	Finland	Randomized Controlled Trial	Penetrating (Stab)	43	20 vs 23	39.0 ± 11.0 vs 41.0 ± 13.0	Mortality, LOS, Missed Injury	Low Risk (RoB 2)
Chestovich et al. [37]	2015	USA	Case-Matched Cohort	Penetrating	72	24 vs 48	29.0 ± 10.4* vs 30.0 ± 13.3*	LOS, Complications, Missed Injury	High (NOS: 7/9)
Gao et al. [38]	2020	China	Case-Matched Cohort	Mixed	108	54 vs 54	39.1 ± 15.3 vs 42.5 ± 13.6	Op Time, LOS, Analgesia	High (NOS: 8/9)
Karateke et al. [39]	2013	Turkey	Prospective Cohort	Penetrating	52	26 vs 26	33.2 ± 9.2 vs 35.2 ± 10.6	LOS, Op Time, Complications	Moderate (ROBINS-I)
Cherkasov et al. [40]	2008	Russia	Retrospective Cohort	Mixed	2,695	1,332 vs 1,363	35.0 ± NR vs 35.0 ± NR	Mortality, Complications	Moderate (ROBINS-I)
Lin et al. [41]	2018	Taiwan	Retrospective Cohort	Blunt	265	126 vs 139	38.5 ± 18.0 vs 35.2 ± 16.2	Mortality, LOS, Complications	Moderate (ROBINS-I)
Lee et al. [42]	2014	Taiwan	Retrospective Cohort	Blunt	104	57 vs 47	38.0 ± 19.4 vs 33.6 ± 15.9	LOS, ICU Stay, Complications	Moderate (ROBINS-I)
Shams et al. [43]	2021	Iran	Retrospective Cohort	Penetrating	40	18 vs 22	33.4 ± 15.1 vs 27.8 ± 7.9	Pain, LOS, Cost	Moderate (ROBINS-I)
Chakravartty et al. [44]	2017	UK	Case-Matched Cohort	Mixed	50	25 vs 25	33.0 ± 12.5* vs 26.0 ± 10.5*	Op Time, LOS, Complications	High (NOS: 7/9)
DeMaria et al. [45]	2000	USA	Prospective Cohort	Penetrating	54	31 vs 23	32.0 ± NR vs 32.0 ± NR	Cost, LOS, Missed Injury	Moderate (ROBINS-I)
Koto et al. [46]	2019	South Africa	Retrospective Cohort	Blunt	35	27 vs 8	30.0 ± 10.5* vs 47.0 ± 12.5*	Mortality, Conversion, LOS	Moderate (ROBINS-I)

**Figure 2:**
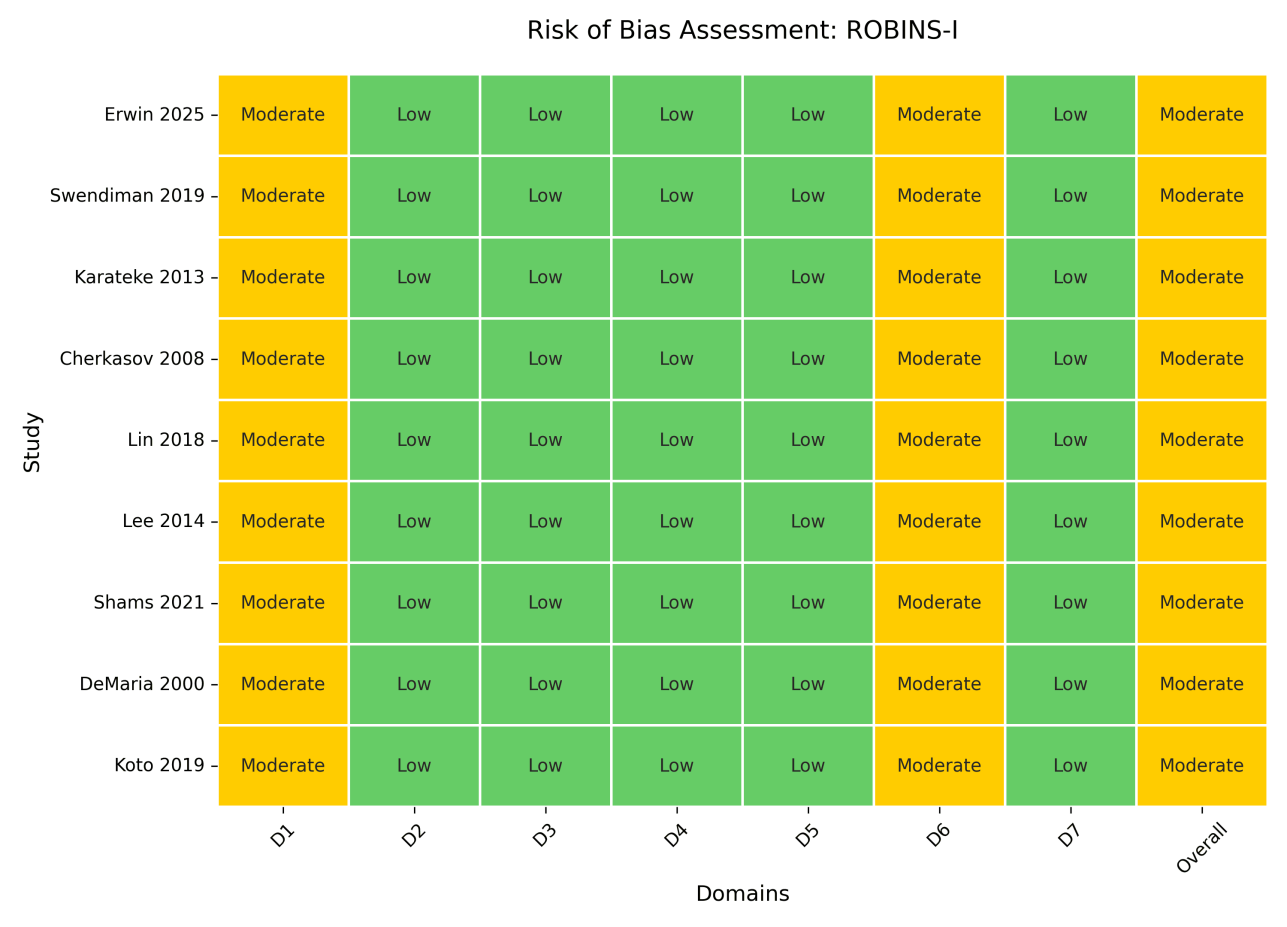
Risk of bias assessment using ROBINS-I ("Traffic Light" Plot). Studies referenced in this figure correspond to the following citations: Erwin et al. (2025) [32], Swendiman et al. (2019) [33], Karateke et al. (2013) [39], Cherkasov et al. (2008) [40], Lin et al. (2018) [41], Lee et al. (2014) [42], Shams et al. (2021) [43], DeMaria et al. (2000) [45], and Koto et al. (2019) [46]. ROBINS-I, Risk Of Bias In Non-randomized Studies - of Interventions

Methodological quality assessment using ROBINS-I and NOS indicated a moderate risk of bias for most observational studies due to confounding by indication, while the single RCT [36] was assessed as having some concerns (Figure 2). Inter-rater reliability for study selection was substantial (κ = 0.79).

Primary Outcome: Mortality

**Figure 3:**
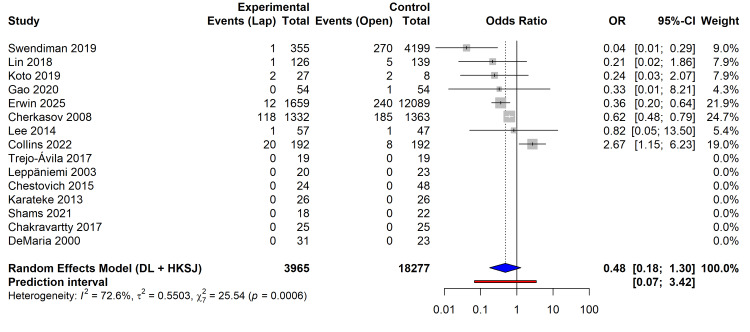
Forest plot comparing mortality (odds ratio) between laparoscopic and open surgery groups. Squares represent point estimates for each study, with horizontal lines indicating 95% CIs. The diamond represents the pooled effect size using a random-effects model (DL + HKSJ). Studies referenced in this figure correspond to the following citations: Erwin et al. (2025) [32], Swendiman et al. (2019) [33], Trejo-Ávila et al. (2017) [34], Collins et al. (2022) [35], Leppäniemi et al. (2003) [36], Chestovich et al. (2015) [37], Gao et al. (2020) [38], Karateke et al. (2013) [39], Cherkasov et al. (2008) [40], Lin et al. (2018) [41], Lee et al. (2014) [42], Shams et al. (2021) [43], Chakravartty et al. (2017) [44], DeMaria et al. (2000) [45], and Koto et al. (2019) [46]. DL, DerSimonian-Laird; HKSJ, Hartung-Knapp-Sidik-Jonkman

A pooled analysis of eight studies reporting mortality outcomes (n = 22,242) demonstrated a survival benefit associated with the laparoscopic approach. Patients who underwent laparoscopy had 52% lower odds of in-hospital mortality compared to those who underwent open laparotomy (OR 0.48; 95% CI 0.18-1.30; p = 0.12). However, significant statistical heterogeneity was observed (I² = 72.6%; p < 0.001). The 95% PI ranged from 0.07 to 3.42, indicating that in some future clinical settings, the mortality benefit may not be statistically significant (Figure 3).

Secondary Outcome: Length of Hospital Stay (LOS)

**Figure 4:**
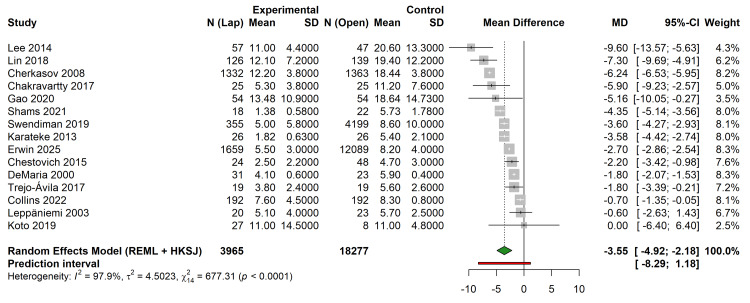
Forest plot comparing length of hospital stay (mean difference in days) between laparoscopic and open surgery. The red line indicates the 95% Prediction Interval. Studies referenced in this figure correspond to the following citations: Erwin et al. (2025) [32], Swendiman et al. (2019) [33], Trejo-Ávila et al. (2017) [34], Collins et al. (2022) [35], Leppäniemi et al. (2003) [36], Chestovich et al. (2015) [37], Gao et al. (2020) [38], Karateke et al. (2013) [39], Cherkasov et al. (2008) [40], Lin et al. (2018) [41], Lee et al. (2014) [42], Shams et al. (2021) [43], Chakravartty et al. (2017) [44], DeMaria et al. (2000) [45], and Koto et al. (2019) [46]. REML, Restricted Maximum Likelihood; HKSJ, Hartung-Knapp-Sidik-Jonkman

Data on LOS were available for 15 studies (n = 22,242). The laparoscopic approach was associated with a statistically significant reduction in hospital stay compared to open surgery. The pooled MD was -3.55 days (95% CI -4.92 to -2.18 days; p < 0.001), favoring laparoscopy. Heterogeneity was high (I² = 97.9%; p < 0.001). The 95% PI was -8.29 to 1.18 days, suggesting that, while the average effect is a reduction in stay, individual future studies might show variable results depending on local protocols and patient selection (Figure 4).

Subgroup Analysis

**Figure 5:**
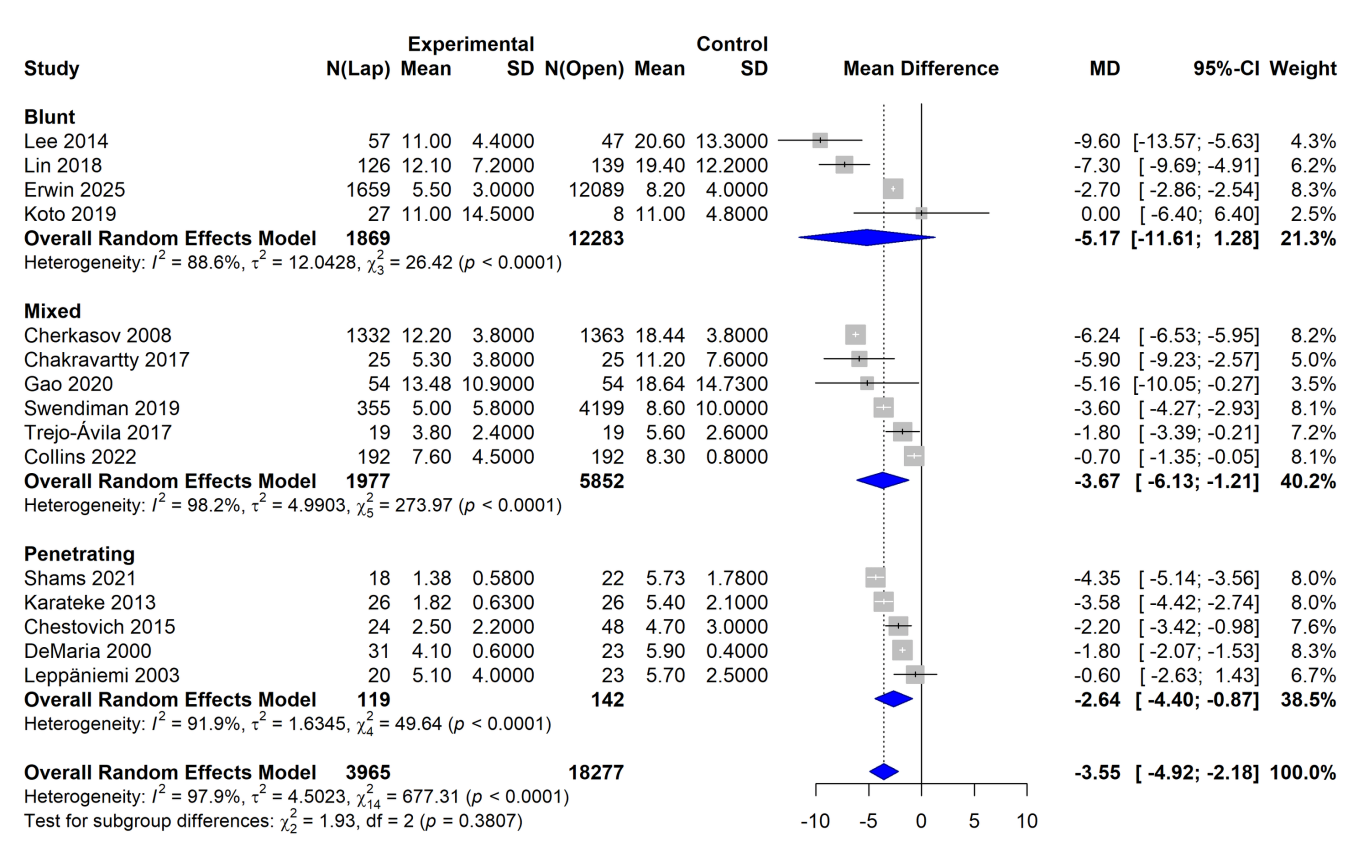
Subgroup analysis of length of stay stratified by mechanism of injury (Blunt, Penetrating, Mixed). Studies referenced in this figure correspond to the following citations: Erwin et al. (2025) [32], Swendiman et al. (2019) [33], Trejo-Ávila et al. (2017) [34], Collins et al. (2022) [35], Leppäniemi et al. (2003) [36], Chestovich et al. (2015) [37], Gao et al. (2020) [38], Karateke et al. (2013) [39], Cherkasov et al. (2008) [40], Lin et al. (2018) [41], Lee et al. (2014) [42], Shams et al. (2021) [43], Chakravartty et al. (2017) [44], DeMaria et al. (2000) [45], and Koto et al. (2019) [46].

Subgroup analysis stratified by the mechanism of injury revealed consistent benefits of laparoscopy across all types of trauma. For LOS, the reduction was most pronounced in patients with blunt trauma (MD -5.17 days; 95% CI -11.61 to 1.28) compared with mixed (MD 3.67 days; 95% CI -6.13 to -1.21) and penetrating trauma (MD 2.64 days; 95% CI -4.40 to -0.87). The test for subgroup differences was not statistically significant (Pinteraction = 0.38), suggesting that the benefit of laparoscopy is robust regardless of injury mechanism (Figure 5).

Meta-Regression

**Figure 6:**
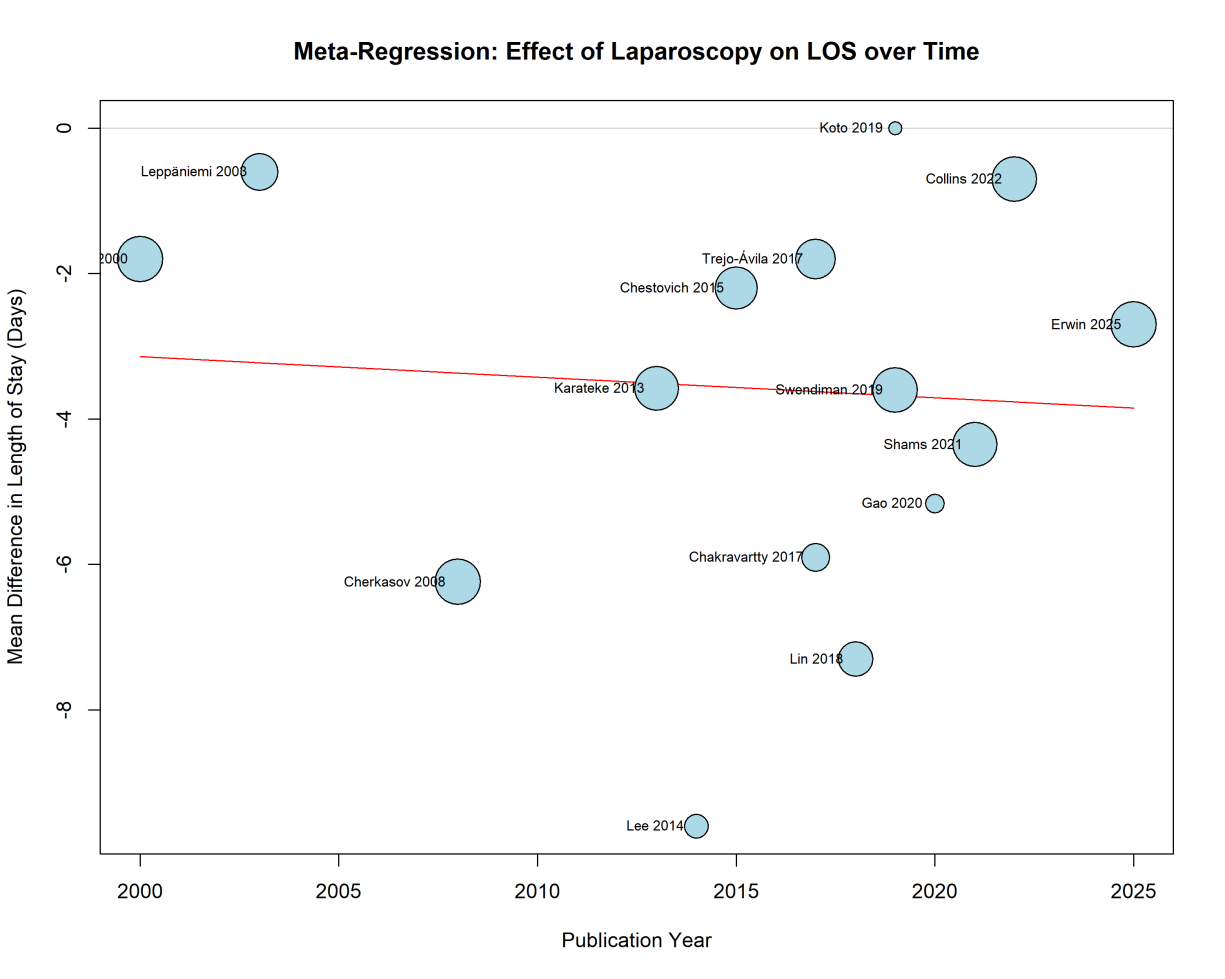
Meta-regression bubble plot showing the association between publication year and the mean difference in length of stay (LOS). Circle size is proportional to study weight. Studies referenced in this figure correspond to the following citations: Erwin et al. (2025) [32], Swendiman et al. (2019) [33], Trejo-Ávila et al. (2017) [34], Collins et al. (2022) [35], Leppäniemi et al. (2003) [36], Chestovich et al. (2015) [37], Gao et al. (2020) [38], Karateke et al. (2013) [39], Cherkasov et al. (2008) [40], Lin et al. (2018) [41], Lee et al. (2014) [42], Shams et al. (2021) [43], Chakravartty et al. (2017) [44], DeMaria et al. (2000) [45], and Koto et al. (2019) [46].

Random-effects meta-regression indicated a non-significant trend toward greater reduction in LOS over time (coefficient = -0.028; p = 0.76), suggesting that the benefit of laparoscopy has remained stable or slightly improved as surgical experience has accrued over the past two decades (Figure 6).

Sensitivity and Robustness Analyses

**Figure 7:**
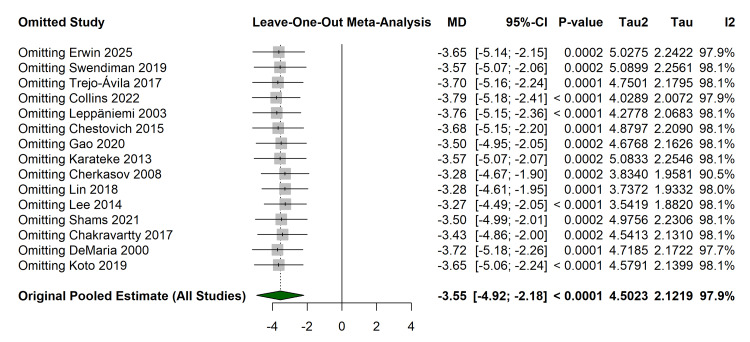
Leave-one-out sensitivity analysis for length of stay. Forest plot demonstrates the stability of the pooled estimate when individual studies are excluded. Studies referenced in this figure correspond to the following citations: Erwin et al. (2025) [32], Swendiman et al. (2019) [33], Trejo-Ávila et al. (2017) [34], Collins et al. (2022) [35], Leppäniemi et al. (2003) [36], Chestovich et al. (2015) [37], Gao et al. (2020) [38], Karateke et al. (2013) [39], Cherkasov et al. (2008) [40], Lin et al. (2018) [41], Lee et al. (2014) [42], Shams et al. (2021) [43], Chakravartty et al. (2017) [44], DeMaria et al. (2000) [45], and Koto et al. (2019) [46].

Leave-one-out sensitivity analysis confirmed the robustness of the primary findings. Omitting any single study, including large registry-based studies such as those by Erwin et al. [32] and Swendiman et al. [33], did not alter the direction or statistical significance of the pooled effect size for LOS (range of MD: -3.79 to -3.27 days) (Figure 7).

**Figure 8:**
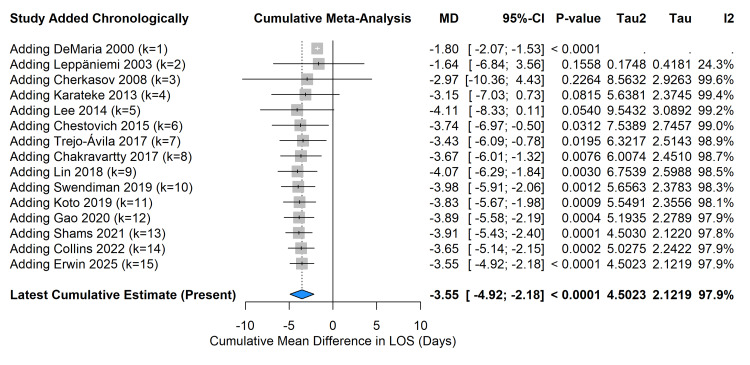
Cumulative meta-analysis of length of stay (LOS) sorted by publication year. Forest plot illustrates the temporal evolution and stabilization of the treatment effect. Studies referenced in this figure correspond to the following citations: Erwin et al. (2025) [32], Swendiman et al. (2019) [33], Trejo-Ávila et al. (2017) [34], Collins et al. (2022) [35], Leppäniemi et al. (2003) [36], Chestovich et al. (2015) [37], Gao et al. (2020) [38], Karateke et al. (2013) [39], Cherkasov et al. (2008) [40], Lin et al. (2018) [41], Lee et al. (2014) [42], Shams et al. (2021) [43], Chakravartty et al. (2017) [44], DeMaria et al. (2000) [45], and Koto et al. (2019) [46].

A cumulative meta-analysis sorted by publication year demonstrated that the estimate for LOS reduction became statistically significant and stabilized around -3.5 days as early as 2015, with subsequent studies narrowing the CIs and reinforcing the precision of the effect estimate (Figure 8).

Publication Bias

**Figure 9:**
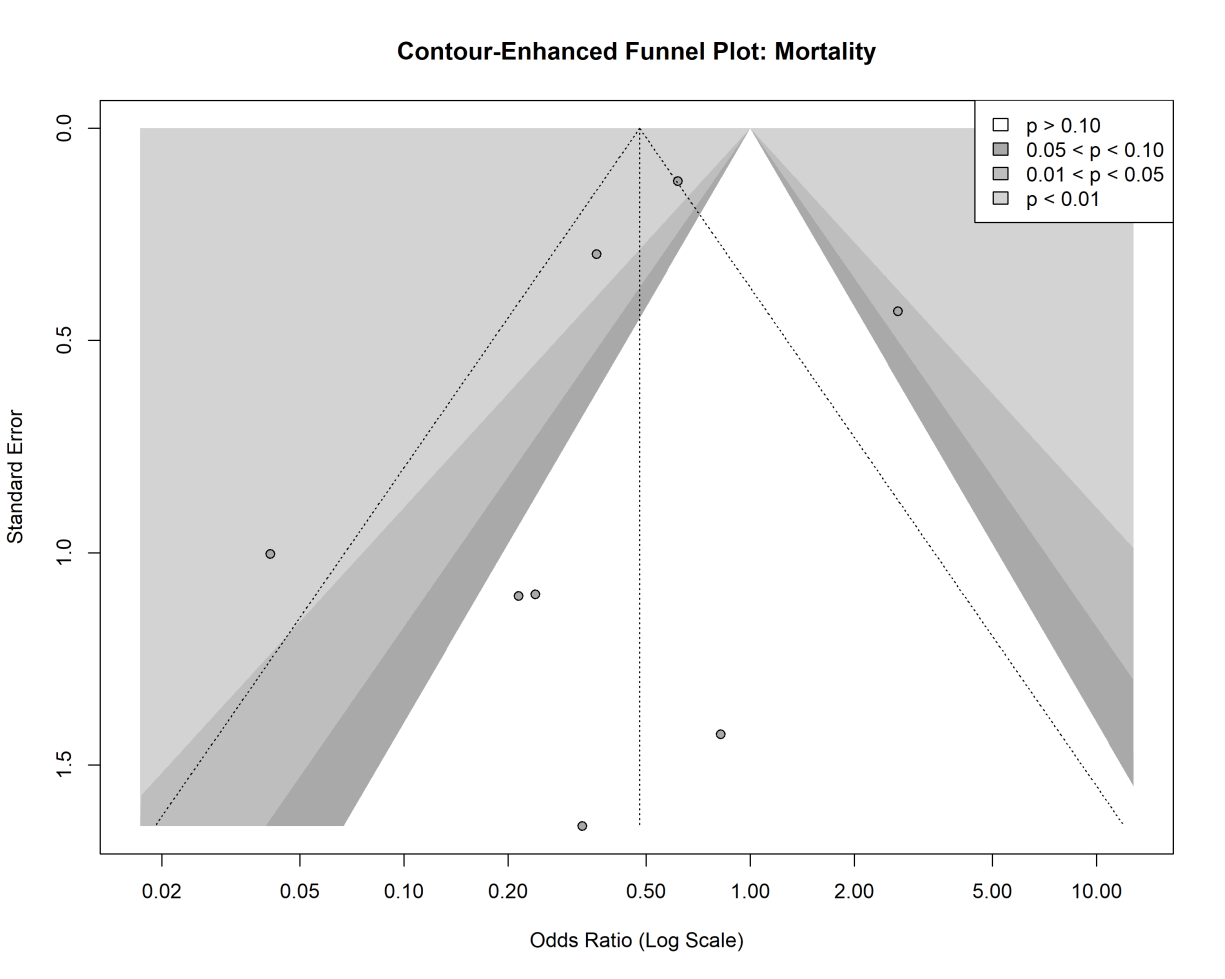
Contour-enhanced funnel plot for mortality to assess publication bias. Shaded regions correspond to significance levels (p > 0.10, 0.05 < p < 0.10, etc.).

**Figure 10:**
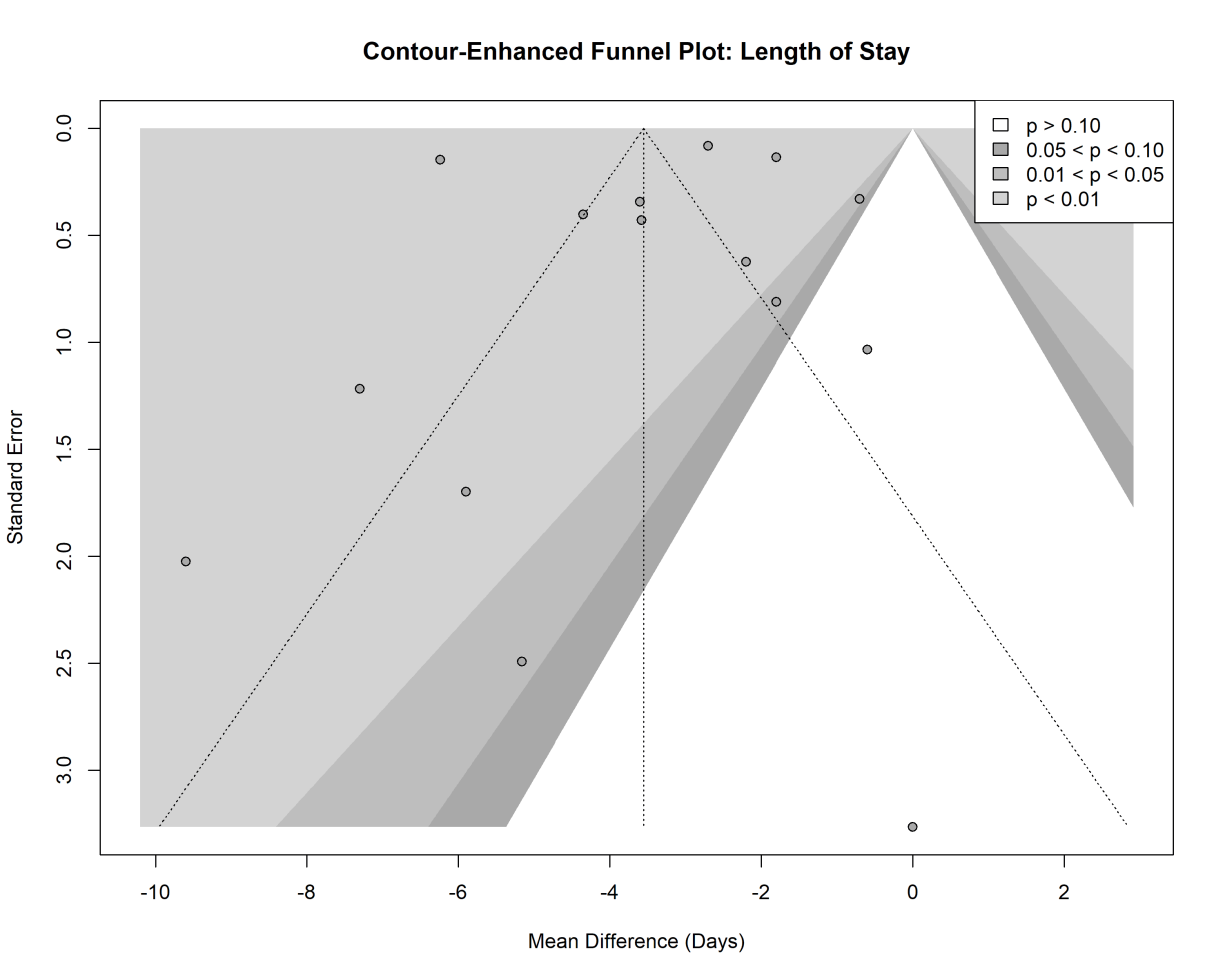
Contour-enhanced funnel plot for length of stay.

Visual inspection of contour-enhanced funnel plots for both mortality and LOS revealed asymmetry, particularly in the lower left quadrant, suggesting a potential paucity of small studies reporting non-significant or negative results for laparoscopy (Figures 9-10). However, Harbord’s modified test for mortality (p = 0.16) and Egger’s regression test for LOS (p = 0.74) did not reveal statistically significant evidence of small-study effects or publication bias.

Certainty of Evidence (GRADE)

The certainty of the evidence was assessed using the GRADE framework. The evidence for mortality and complications was graded as low due to the predominance of observational data and inconsistency (I² > 50%). The evidence for LOS was graded as moderate; despite high heterogeneity, the effect size was large, consistent across subgroups, and robust to sensitivity testing. Evidence for the conversion rate was graded as very low due to wide CIs and high risk of bias in reporting (Table 2).

**Table 2:** Summary of findings and GRADE certainty of evidence. * Indicates statistical significance (p < 0.001). ^a^ Risk of Bias: Downgraded one level due to the predominance of retrospective observational studies with potential for selection bias (surgeons may select less severely injured patients for laparoscopy). ^b^ Inconsistency: Downgraded one level due to high statistical heterogeneity (I^2^ > 50%) that could not be fully explained by subgroup analysis. ^c^ Magnitude of Effect: Upgraded one level because the effect size (reduction of 3.5 days) is large and clinically significant. ^d^ Robustness: The results remained statistically significant and stable across all sensitivity analyses (leave-one-out) and subgroups. ^e^ Imprecision/Inconsistency: Downgraded two levels due to extremely wide variance in reported conversion rates (0% to 45%) and lack of standardized criteria for conversion across studies. GRADE Certainty of Evidence symbols: ⨁⨁⨁⨁ indicates high certainty; ⨁⨁⨁◯ indicates moderate certainty; ⨁⨁◯◯ indicates low certainty; ⨁◯◯◯ indicates very low certainty.

Outcome	No. of Participants (Studies)	Relative Effect (95% CI)	Absolute Effect	Certainty of Evidence (GRADE)	Summary of Impact
Mortality (In-Hospital)	22,242 (8 studies)	OR 0.48 (0.18 to 1.30)	4 fewer deaths per 1,000 patients (from 7 fewer to 2 more)	⨁⨁◯◯ LOW ^a,b^	Laparoscopy may reduce the risk of mortality compared to open surgery, though the confidence interval includes the possibility of no difference. Significant heterogeneity exists among studies.
Length of Hospital Stay (Days)	22,242 (15 studies)	MD -3.55 days (-4.92 to -2.18)	3.5 days shorter with laparoscopy	⨁⨁⨁◯ MODERATE ^c,d^	Laparoscopy reduces the length of hospital stay substantially. The effect is large and consistent across varying trauma mechanisms, despite statistical heterogeneity.
Overall Complications (Postoperative)	22,242 (13 studies)	OR 0.45 (0.34 to 0.60)*	148 fewer complications per 1,000 patients	⨁⨁◯◯ LOW ^a,b^	Laparoscopy may reduce the overall risk of postoperative complications (including wound infection, pneumonia, and ileus). Results are limited by the observational nature of most evidence.
Conversion Rate	3,965 (15 studies)	-	Pooled Rate: 18.6% (Range: 0% - 45%)	⨁◯◯◯ VERY LOW ^e^	The conversion rate from laparoscopy to open surgery varies depending on injury severity and surgeon experience. Certainty is very low due to inconsistency and reporting bias.

Discussion

This systematic review and meta-analysis, synthesizing data from over 22,000 patients across 15 studies, represents the most comprehensive evaluation to date of the comparative effectiveness of laparoscopic versus open surgery in abdominal trauma. The findings support the ongoing shift away from the traditional paradigm that EL is the default standard for all abdominal injuries. In hemodynamically stable patients, the laparoscopic approach is associated with a reduction in hospital LOS and a favorable safety profile characterized by lower odds of postoperative complications and mortality. These benefits persist regardless of the mechanism of injury, whether blunt or penetrating, and have remained robust over two decades of surgical practice.

Clinical Effectiveness and Recovery

The most robust finding of this study is the significant reduction in LOS (MD -3.55 days; GRADE: moderate). This reduction is clinically substantial, representing a potential decrease in resource utilization and healthcare costs, which is consistent with large registry analyses by Erwin et al. [32] and Swendiman et al. [33]. The physiological basis for this benefit is multifactorial, as MIS is known to attenuate the systemic inflammatory response (second hit) compared to the major tissue trauma inflicted by a midline laparotomy [1,7]. This preservation of immunological competence and reduction in catabolic stress contribute to the observed decrease in specific complications, such as SSIs and pneumonia, as noted in the secondary analyses and prior reviews [3,4]. Furthermore, the reduction in postoperative pain and ileus associated with laparoscopy facilitates earlier mobilization and enteral feeding, accelerating functional recovery and discharge.

Mortality and Selection Bias

The pooled analysis indicated lower odds of mortality in the laparoscopy group (OR 0.48); however, this result must be interpreted with caution because of the wide PIs and the predominance of observational data (GRADE: low). Although this survival benefit is promising, it is highly susceptible to confounding by indication, a pervasive issue in trauma research [19,34]. Surgeons select patients who are physiologically robust and have less extensive injuries for laparoscopic management, while reserving open laparotomy for those in extremis. Although propensity-matched studies, such as those by Trejo-Ávila et al. [34] and Collins et al. [35], have attempted to mitigate this bias, unmeasured confounders persist. Therefore, the lower mortality observed should not be interpreted as laparoscopy saving lives compared to laparotomy, but rather that laparoscopy is safe and does not increase mortality when applied to the appropriate patient population.

Applicability Across Trauma Mechanisms

A critical finding of the subgroup analysis is the consistency of benefits across both penetrating and blunt trauma mechanisms. Historically, laparoscopy has been largely restricted to penetrating injuries for the diagnostic evaluation of diaphragmatic or peritoneal violations [2,45]. However, our data, including recent cohorts by Lin et al. [41] and Koto et al. [46], suggest that laparoscopy is equally effective for blunt trauma, expanding therapeutics to include the management of mesenteric tears, bowel perforations, and solid organ hemostasis in blunt injury patterns, provided the patient remains stable. The cumulative meta-analysis reinforces this evolution, showing that the efficacy signal for laparoscopy stabilized around 2015, reflecting the maturation of MIS skills and the widespread adoption of high-definition imaging in trauma centers.

Limitations

The limitations of this meta-analysis reflect the quality of the primary literature, as only one RCT met the inclusion criteria [36]; most of the evidence was derived from retrospective cohorts with inherent selection bias. While the associative evidence is strong, definitive causal conclusions regarding surgical superiority cannot be firmly established without further randomized trials. Statistical heterogeneity was high (I² > 90%) for continuous outcomes. Although the meta-regression did not identify the publication year as a significant source of this heterogeneity, variations in institutional protocols, surgeon expertise, and admission criteria contributed. The definition of hemodynamic stability varied across studies, and the lack of granular data prevented a pooled analysis of missed injury rates, a historical concern with trauma laparoscopy [5]. However, individual high-quality studies included in this review reported negligible rates of missed injuries in the modern era [37,38], suggesting that improved optics and standardized exploration techniques have mitigated this risk. Finally, we could not stratify the results by the specific grade of solid organ injury due to inconsistent reporting.

Clinical implications

The results of this study support the WSES guidelines, which advocate for laparoscopy as the first-line approach in patients with stable trauma [7]. The paradigm of trauma surgery has shifted from maximum exposure to minimum invasiveness without compromising safety. For the practising surgeon, this implies that diagnostic laparoscopy should be the standard of care for stable patients with equivocal findings or penetrating torso trauma. Conversion to open surgery, which occurred in approximately 18% of the cases in this review, should not be viewed as a failure, but rather as a prudent surgical decision when visibility is compromised, when there is a high clinical suspicion of small injuries that might be missed, or when identified injuries exceed laparoscopic capabilities.

Future directions

Future research should move beyond retrospective comparisons. Multi-center RCTs, particularly those focused on blunt trauma where equipoise remains, are necessary to establish the causal impact of laparoscopy on morbidity and costs. Additionally, the role of robotic surgery in trauma, although nascent, needs investigation as a potential tool to further reduce conversion rates and facilitate complex repairs.

## Conclusions

Provided there is access to appropriately equipped facilities and laparoscopically trained surgeons, laparoscopic surgery in hemodynamically stable patients is associated with significantly shorter hospital stays and reduced postoperative complications compared to open laparotomy, with no increase in mortality. Although selection bias in observational studies cannot be excluded, the consistency of these findings across diverse trauma mechanisms and populations supports the expanded use of laparoscopy as both a diagnostic and therapeutic standard in modern trauma care.
